# Development of *Beet necrotic yellow vein virus*‐based vectors for multiple‐gene expression and guide RNA delivery in plant genome editing

**DOI:** 10.1111/pbi.13055

**Published:** 2019-01-17

**Authors:** Ning Jiang, Chao Zhang, Jun‐Ying Liu, Zhi‐Hong Guo, Zong‐Ying Zhang, Cheng‐Gui Han, Ying Wang

**Affiliations:** ^1^ State Key Laboratory for Agro‐biotechnology and Ministry of Agriculture Key Laboratory of Pest Monitoring and Green Management College of Plant Protection China Agricultural University Beijing China; ^2^ College of Chemistry Biology and Environment Yuxi Normal University Yuxi China

**Keywords:** *Beet necrotic yellow vein virus*, multiple‐genes expression vector, sugar beet, guide RNA delivery, genome editing

## Abstract

Many plant viruses with monopartite or bipartite genomes have been developed as efficient expression vectors of foreign recombinant proteins. Nonetheless, due to lack of multiple insertion sites in these plant viruses, it is still a big challenge to simultaneously express multiple foreign proteins in single cells. The genome of *Beet necrotic yellow vein virus* (BNYVV) offers an attractive system for expression of multiple foreign proteins owning to a multipartite genome composed of five positive‐stranded RNAs. Here, we have established a BNYVV full‐length infectious cDNA clone under the control of the *Cauliflower mosaic virus* 35S promoter. We further developed a set of BNYVV‐based vectors that permit efficient expression of four recombinant proteins, including some large proteins with lengths up to 880 amino acids in the model plant *Nicotiana benthamiana* and native host sugar beet plants. These vectors can be used to investigate the subcellular co‐localization of multiple proteins in leaf, root and stem tissues of systemically infected plants. Moreover, the BNYVV‐based vectors were used to deliver *NbPDS* guide RNAs for genome editing in transgenic plants expressing Cas9, which induced a photobleached phenotype in systemically infected leaves. Collectively, the BNYVV‐based vectors will facilitate genomic research and expression of multiple proteins, in sugar beet and related crop plants.

## Introduction

In the 1980s, *Cauliflower mosaic virus* (CaMV) and *Brome mosaic virus* (BMV) were engineered as the first DNA and RNA plant virus vectors to express bacterial genes (Brisson *et al*., 1984; French *et al*., 1986). Since then, plant virus‐based vectors have been widely used as effective tools for recombinant protein expression and genomic research (Palmer and Gleba, 2014), especially for some plant species that are difficult to transform. So far, a number of plant viruses have been developed as delivery vectors for multiple purposes, such as *tobamoviruses* (Takamatsu *et al*., 1987; Yusibov *et al*., 1999)*, potexviruses* (Baulcombe *et al*., 1995; Chapman *et al*., 1992; Zhang *et al*., 2013)*, Potyvirus* (Jarugula *et al*., 2016; Lellis *et al*., 2002; Majer *et al*., 2015; Seo *et al*., 2016), *Comoviruses* (Sainsbury *et al*., 2008; Zhang *et al*., 2010)*, Geminiviruses* (Stanley, 1993)*, Caulimovirus (Brisson et al.,* 1984 *)* and *Necrovirus* (Zhou *et al*., 2010) in dicotyledonous plants, *Barley stripe mosaic virus* (Cheuk and Houde, 2018; Lee *et al*., 2012; Yuan *et al*., 2011)*, Soil‐borne wheat mosaic virus* (Jarugula *et al*., 2016), *Wheat steak mosaic virus* (Choi *et al*., 2000), and *Foxtail mosaic virus* (Bouton *et al*., 2018) in monocotyledonous plants. However, some studies in basic and applied plant biology needing expression of functional complex heterologous proteins, production of antibodies and pharmaceutical peptides, require simultaneous expression of two or more genes within single cells. However, the plant virus‐based vectors described above are unable to stably express multiple proteins due to lack of multiple insertion sites. In addition, the size constraints of protein expression by these virus vectors prohibit stable expression of large foreign proteins. Hence, it is important to develop some efficient and user‐friendly plant viral‐based systems for concurrent expression of multiple‐genes.

*Beet necrotic yellow vein virus* (BNYVV), transmitted by *Polymyxa betae* (*P. betae*), is a member of the genus *Benyvirus* with four or five single‐stranded RNA genomes (Rush, 2003). RNA1 and RNA2, encoding “house‐keeping” genes, are sufficient for the viral life cycle in some experimental host plants, such as *Tetragania expansa* (*T. expansa*), *Chenopodium quinoa* (*C. quinoa*)*, Spinacea oleracea* and *Nicotiana benthamiana (N. benthamiana)* (McGrann *et al*., 2009; Richards and Tamada, 1992). RNA3, RNA4 and RNA5 play important roles in the natural infection processes (McGrann *et al*., 2009). RNA3‐encoded p25 and RNA5‐encoded p26 are pathogenicity or synergistic in a synergistic way on *T. expansa*,* Chenopodium and Beta* species (Jupin *et al*., 1992; Lauber *et al*., 1998; Link *et al*., 2005), while RNA4‐encoded p31 is associated with efficient vector transmission and root‐specific RNA silencing suppression (Andika *et al*., 2005; Rahim *et al*., 2007). In some isolates or during serial mechanical passages, RNA2, RNA3, RNA4 and RNA5 undergo internal deletions in the coding regions, indicating these regions are not essential for RNA replication (Bouzoubaa *et al*., 1985, 1991; Koenig *et al*., 1986; Tamada and Kusume, 1991). Moreover, each of RNA2, RNA3, RNA4 and RNA5 contains an insertion site, suggesting that BNYVV has great potential for development as a multiple‐genes expression vector.

Full‐length infectious cDNA clones of BNYVV including B‐type isolate F‐13 and A‐type isolate Yu2 under the control of the T7 and CaMV 35S promoter have been constructed for reverse genetics analysis of BNYVV (Delbianco *et al*., 2013; Laufer *et al*., 2018a). RNA3‐ and RNA5‐based replicons have been constructed for foreign gene expression in *Beta macrocarpa*,* C. quinoa* and *N. benthamiana* (Alice *et al*., 2013; Delbianco *et al*., 2013; Erhardt *et al*., 2000; Laufer *et al*., 2018b; Schmidlin *et al*., 2005). Recently, portions of the RNA2 read‐through domain (RTD) of BNYVV and *Beet soil‐borne mosaic virus* (BSBMV) were substituted by fluorescent reporter genes to investigate co‐ and superinfection of these two viruses in *N. benthamiana* (Laufer *et al*., 2018a). However, BNYVV RNA4 has not been developed as an expression vector due to the complex involvement of the cis‐essential replication sequences that are located in RNA4. In addition, the capacity of BNYVV‐derived vectors for multiple‐gene expression (up to four genes) and functional gene studies have some problems. For example, both RNA1 and RNA2 from isolate F‐13 and Yu2 induce severe symptoms such as light yellow chlorosis and leaf crinkling on *N. benthamiana* (Delbianco *et al*., 2013; Laufer *et al*., 2018a), which might disturb the functional analysis of candidate genes. However, the Hu3 Chinese BNYVV isolate derived from isolate Hu only induces very mild symptoms in *N. benthamiana* (Wang *et al*., 2011), indicating that isolate Hu3 has substantial potential for vector development.

In this study, we constructed infectious cDNA clones of Hu3 isolate under the control of the CaMV 35S promoter. We further engineered Hu3 cDNA clones to generate multi‐gene expression vectors for expression of four reporter proteins in *N. benthamiana* and *Beta* species. Moreover, the BNYVV‐based vectors were successfully used to express multiple proteins for co‐localization assays and guide RNAs for plants genome editing assays.

## Results

### Construction of full‐length infectious cDNA clones of BNYVV isolate Hu

**Figure 1:**
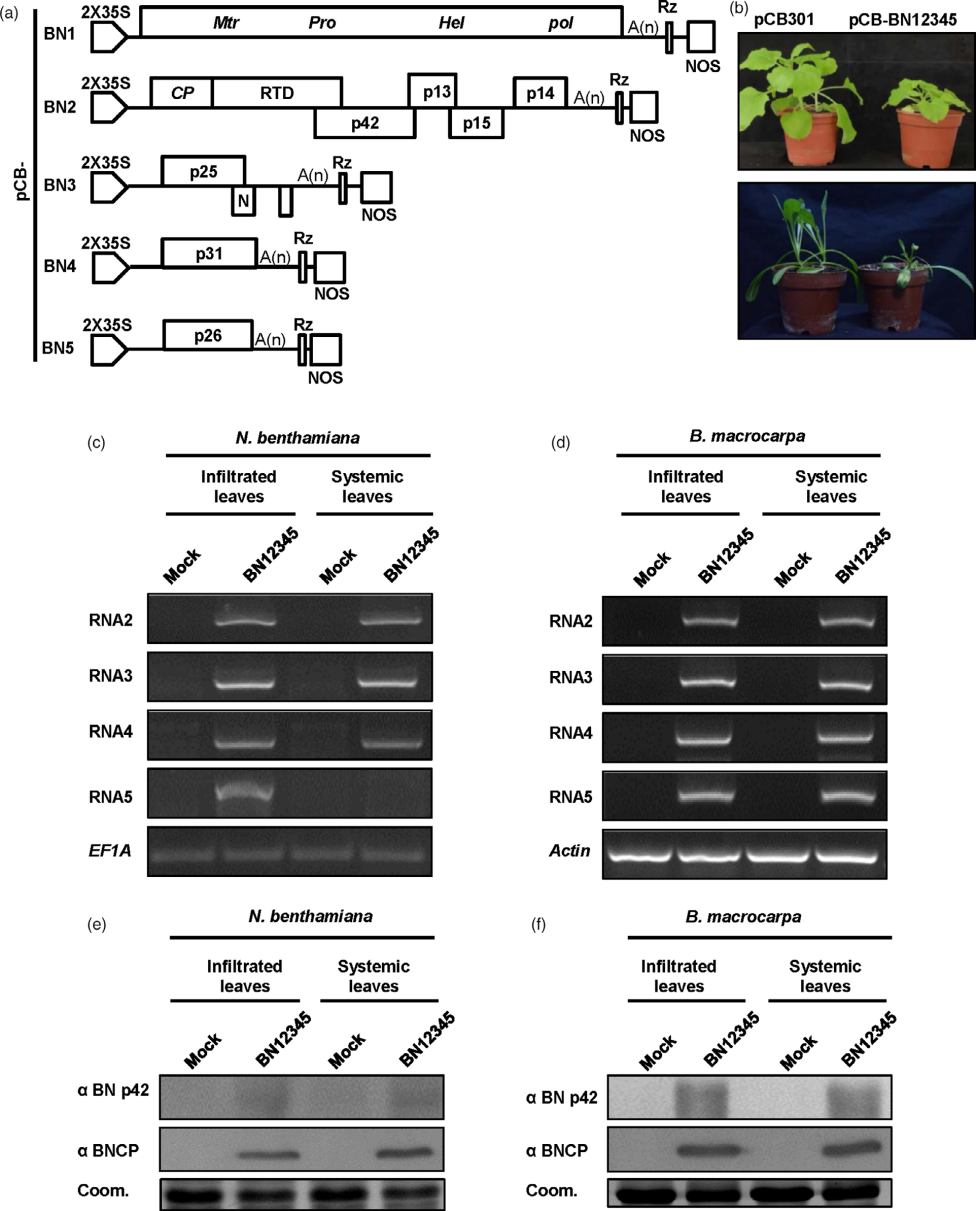
Testing the infectivity of BNYVV full‐length cDNA clones in *Nicotiana benthamiana* and *Beta macrocarpa*. (a) Schematic representation of the construction of BNYVV full‐length infectious clones. BNYVV RNA1, RNA2, RNA3, RNA4 and RNA5 were cloned between the double CaMV 35S promoter (2 × 35S) and a ribozyme sequence (Rz) followed by a Nos terminator (Nos) in the pCB301 plasmid. (b) Systemic symptoms on *N. benthamiana* and *B. macrocarpa* agroinfiltrated with *Agrobacterium tumefaciens* cells harbouring the pCB‐BN1, pCB‐BN2, pCB‐BN3, pCB‐BN4 and pCB‐BN5 (pCB‐BN12345), or the pCB301 empty vector. (c) and (d): RT‐PCR Detection of BNYVV RNAs 2, 3, 4 and 5 from infiltrated and systemically infected leaves of *N. benthamiana* (c) and *B. macrocarpa* (d). The Mock and BN12345 DNAs were obtained by RT‐PCR from pCB301 and pCB‐BN12345‐infiltrated plants, respectively. *N. benthamiana EF1A* and *B. macrocarpa Actin* genes were used as loading controls. (e) and (f) Western blotting analysis of BNYVV CP and p42 on both of the infiltrated and systemically infected leaves of *N. benthamiana* (e) and *B. macrocarpa* (f). Coomassie brilliant blue (Coom.) staining is shown as a loading control.

RNA1 and RNA2 encoding “house‐keeping” genes are sufficient for the BNYVV life cycle. To establish infectious clones of the Hu isolate, full‐length cDNA sequences of RNA1 and RNA2 were amplified and cloned between the *Stu*I and *Sma*I sites of the binary vector pCB301‐2x35S‐MCS‐HDV_RZ_‐NOS (hereafter noted as pCB301) (Yao *et al*., 2011) to generate pCB‐BN1 and pCB‐BN2 (Figure 1a). The amplified full‐length cDNA sequences of RNA3/4/5 were inserted between the *Stu*I and *Xba*I sites to produce pCB‐BN3, pCB‐BN4 and pCB‐BN5, respectively (Figure 1a). All clones were identified by sequencing and then transferred into *Agrobacterium tumefaciens* cells (strain C58CI).

To verify infectivity of the BNYVV full‐length cDNA clones, fully expanded leaves of *N*. *benthamiana* and *B. macrocarpa* were infiltrated with bacterial cultures carrying an empty vector or a mixture of pCB‐BN1, pCB‐BN2, pCB‐BN3, pCB‐BN4 and pCB‐BN5 (pCB‐BN12345). Consistent with previous studies about viral total RNA infection (Delbianco *et al*., 2013; Wang *et al*., 2011), the pCB‐BN12345‐infiltrated *N. benthamiana* leaves exhibited chlorotic spots at 5 days post‐infiltration (dpi) (Figure S1), while the infiltrated *B. macrocarpa* leaves developed yellow lesions at 7 dpi (Figure S1). Subsequently, compared with mock inoculated plants, infiltration of pCB‐BN12345 induced strong systemic dwarfing and curling symptoms on leaves of both *N. benthamiana* and *B. macrocarpa* by 10 and 14 dpi (Figure 1b), respectively. In three independent experiments, the systemic symptoms induced by pCB‐BN12345 were repeatedly in agreement with those induced by isolate Hu as reported previously (Wang *et al*., 2011).

We further performed RT‐PCR detection to confirm the presence of individual BNYVV RNAs in the pCB‐BN12345‐infected leaves. To this end, total RNAs of infiltrated and systemically infected leaves of *N. benthamiana* and *B. macrocarpa* were extracted for RT‐PCR detection with specific primers targeting BNYVV RNA2‐5 as shown in Table S1. The results showed that RNA2, 3, 4 could be detected in both the infiltrated and systemic infected *N. benthamiana* leaves by pCB‐BN12345 (Figure 1c). By contrast, RNA5 was only detected in the infiltrated leaves (Figure 1c), but disappeared in most systemically infected leaves of *N. benthamiana* (Figure 1c), which indicated that the systemic movement of RNA5 was inefficient in *N. benthamiana* plants as reported previously (Wang *et al*., 2011). Nonetheless, RNA2, 3, 4 and 5 were individually detected in both infiltrated and systemically infected leaves of *B. macrocarpa* plants (Figure 1d), suggesting that all five components are involved in natural infections of BNYVV in *Beta* species.

Western blotting with specific antibodies detected the expression of the coat protein and p42 in infiltrated and systemically infected leaves of *N. benthamiana* (Figure 1e) and *B. macrocarpa* (Figure 1f). Collectively, these results demonstrate that the cDNA clones of pCB‐BN12345 are infectious in *N. benthamiana* and *B. macrocarpa* plants.

### Engineering of BNYVV as a multiple‐gene expression vector

In some BNYVV isolates or during serial mechanical passages, RNA2, RNA3, RNA4 and RNA5 undergo internal deletions in the coding regions, indicating these regions are not essential for RNA replication (Bouzoubaa *et al*., 1985, 1991; Koenig *et al*., 1986; Tamada and Kusume, 1991). Hence, these regions are ideal candidate regions for replacement by foreign genes.

**Figure 2:**
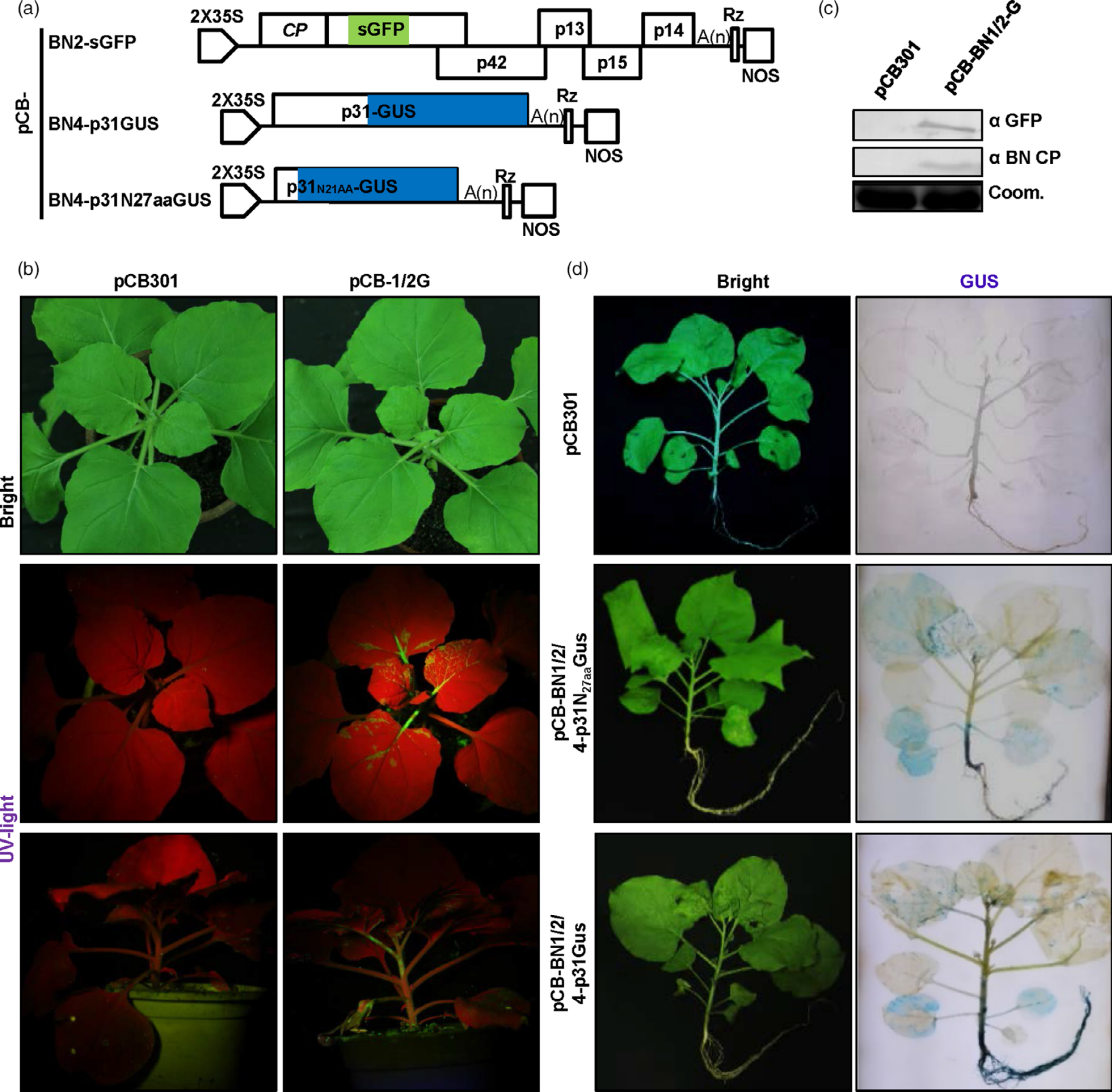
Development of BNYVV RNA2 and RNA4 expression vectors. (a) Schematic representation of the construction of BNYVV RNA2 and RNA4‐based expression vectors. The RTD region from nt 1565 to nt 1872 of RNA2 was replaced by the sGFP. GUS sequence was fused to the C‐terminal of RNA4 encoded p31 or the N‐terminal 27 amino acids of p31. (b) Systematic symptoms (under visible light) and GFP expression pattern (under UV light) of *Nicotiana benthamiana* agroinfiltrated by pCB301 empty vector and pCB‐BN1/2G at 11 dpi, respectively. (c) Immunodetection using antibodies against BNYVV CP and sGFP in *N. benthamiana* systemically infected leaves at 11 dpi using the corresponding antibodies. Coomassie brilliant blue staining (Coom.) is shown as a loading control. (d) Systemic expression of a large protein by the BNYVV RNA4‐based vector after agroinfiltration of *N. benthamiana* plants for expression of pCB‐BN1/2/4p31N27aa‐GUS or pCB‐BN1/2/4‐GUS. GUS activity in the entire plants was detected by histochemical staining with X‐Gluc at 11 dpi. The empty vector pCB301 was used as a negative control.

Given that the *P. betae*‐transmission‐related read‐through domain (RTD) region within BNYVV RNA2 is not essential for RNA replication, we first substituted the RTD region of RNA2 with open reading frames (ORFs) of foreign genes for vector engineering. Firstly, the RTD region from nt1565 to nt1872 of pCB‐BN2 was replaced with a linker consisting of two unique restriction sites (*Nco*I and *Xho*I) to generate pCB‐BN2NX, which facilitates restriction enzyme‐based insertions of genes of interest (Figure S2). Then, the ORF of the green fluorescent protein (GFP) gene was inserted between *Nco*I and *Xho*I sites of pCB‐BN2NX to produce pCB‐BN2‐sGFP (Figure 2a).

To assess the infectivity of pCB‐BN2‐sGFP and the GFP expression efficiency, Agrobacterium harbouring pCB‐BN1 and pCB‐BN2‐sGFP (pCB‐BN1/2‐G) plasmids were mixed and co‐infiltrated into *N. benthamiana* leaves. In addition, the empty vector pCB301 was infiltrated into leaves as a negative control. At 5 dpi, leaves infiltrated with pCB‐BN1/2‐G but not pCB301 developed typical chlorotic spots. However, newly emerging leaves of plants infiltrated with pCB‐BN1/2‐G exhibited no obvious symptoms compared with mock‐treated plants at 11 dpi (Figure 2b, upper panel). However, when these plants were observed under a long wave UV light, the green fluorescence was observed in systemically infected leaves, petioles and stems of the plants infiltrated with pCB‐BN1/2‐G, but not on those of mock‐treated plants (Figure 2b, middle and bottom panels). Furthermore, Western blotting revealed accumulation of virus‐encoded coat protein and GFP (Figure 2c). These results clearly show that BN2‐sGFP can efficiently replicate and move systemically in *N. benthamiana*, and indicate that the cDNA of BNYVV RNA2 allows insertion of heterologous genes in the RTD region.

Then, we engineered RNA4‐derived expression vectors using the Seamless Assembly method. To this end, foreign genes were fused to the C‐terminal of p31 with the porcine teschovirus‐1 2A (P2A) peptide sequence as a linker (Szymczak‐Workman *et al*., 2012). Alternatively, foreign genes were inserted downstream of the first N‐terminal 27 amino acids (N_27aa_) of p31 which contained an essential *cis‐*acting replication element (Figure S2). In this case, β‐glucuronidase (GUS) was fused to the C‐terminus of p31 or inserted downstream of N_27aa_ of p31 to generate pCB‐BN4‐p31GUS and pCB‐BN4‐p31N_27aa_GUS, respectively (Figure 2a).

Subsequently, Agrobacterium harbouring plasmids pCB‐BN4‐p31GUS or pCB‐BN4‐p31N_27aa_GUS were mixed with pCB‐BN1 plus pCB‐BN2 and co‐infiltrated into *N. benthamiana* leaves, respectively. At 11 dpi, these *N. benthamiana* plants were collected for GUS activity staining. As expected, GUS expression was observed in the systemically infected leaves, stems and roots of plant inoculated with pCB‐BN1/2/4‐p31GUS and pCB‐BN1/2/4‐p31_N27aa_GUS, but not in tissues from mock‐treated plants (Figure 2d). It is striking to note that the GUS expression levels in roots infected with pCB‐BN1/2/4‐p31GUS was significantly higher than those infected with pCB‐BN1/2/4‐p31N_27aa_GUS. These results imply that the P31 protein has significant roles in root infections, which is in line with a previous report that p31 is a strong RNA silencing suppressor in roots (Rahim *et al*., 2007). Altogether, our results demonstrate that BNYVV RNA4 can be modified as a vector to successfully express foreign proteins of up to 880 amino acids (p31GUS fusion protein) throughout *N. benthamiana* plants.

**Figure 3:**
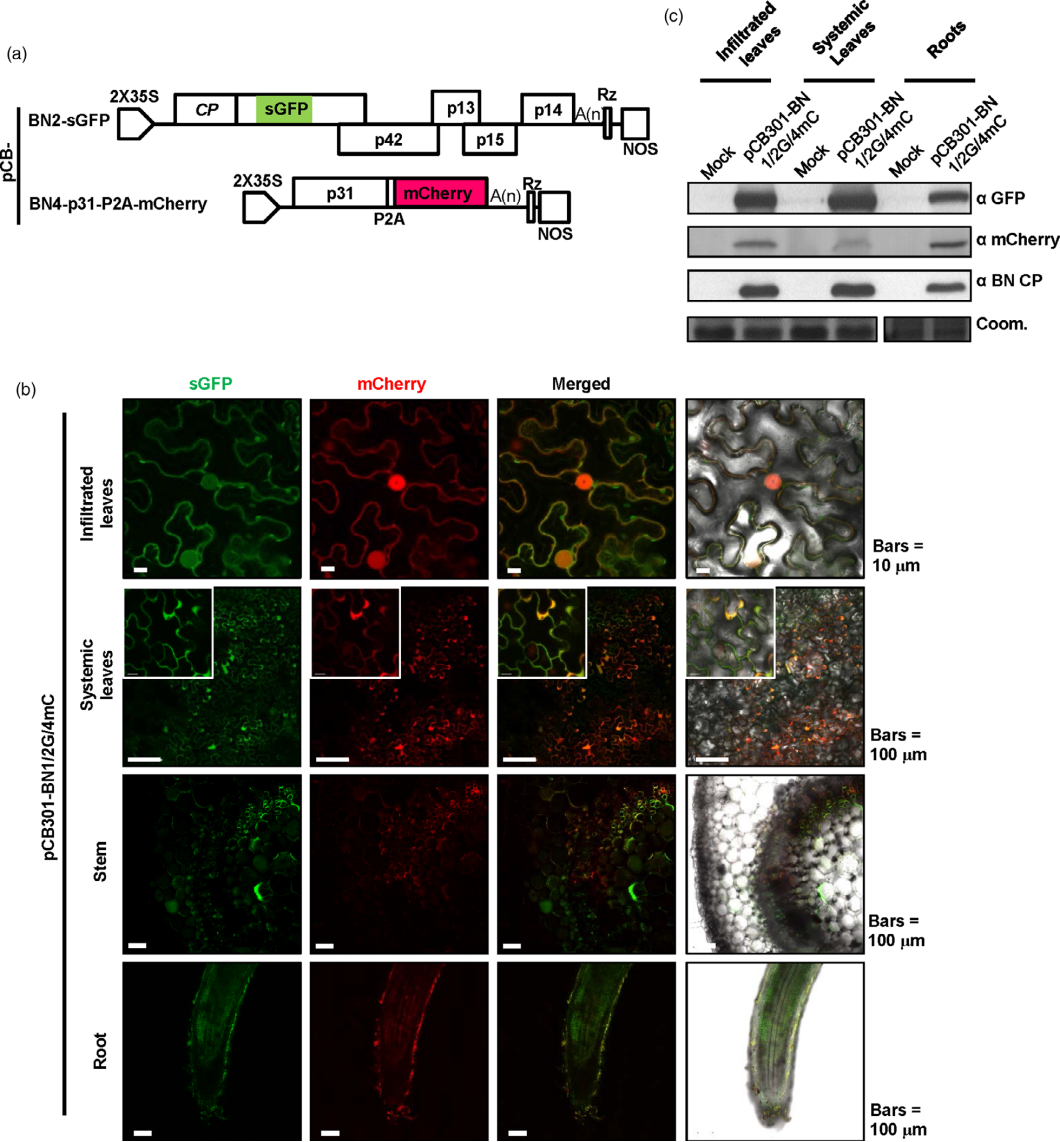
RNA2 and RNA4‐based vectors elicit co‐expression of two proteins in whole plants. (a) Schematic representation of the construction of pCB‐BN2‐sGFP and pCB‐BN4‐p31‐P2A‐mCherry expression vectors. P2A: The “self‐cleaving” 2A sequence of porcine teschovirus‐1. (b) Immunodetection of BNYVV CP, sGFP and mCherry in infiltrated leaves, systemically infected leaves, and roots of *Nicotiana benthamiana* infected with pCB‐BN1/2G/4 m. *Nicotiana benthamiana* plants agroinfiltrated with pCB301 empty vector were used as a negative control. Equal loading was verified by staining the gel with Coom. (c) Confocal microscopy of different tissues of systemically infected *N. benthamiana*. Images of infiltrated leaves were taken at 5 dpi, while those of stem, roots and systemically infected leaves were taken at 14 dpi.

We further examined whether RNA2 and RNA4‐derived vectors could express foreign genes in single cells of infected *N. benthamiana* plants. The mCherry ORF was fused to the C‐terminus of p31 to generate pCB‐BN4‐p31‐P2A‐mCherry (Figure 3a). *Nicotiana benthamiana* plants were infiltrated with *A. tumefaciens* cultures containing combinations of pCB‐BN1, pCB‐BN2‐sGFP plus pCB‐BN4‐p31‐P2A‐mCherry (pCB‐BN1/2‐G/4‐mC) and monitored by confocal laser‐scanning microscopy (CLSM). At 5 dpi, both sGFP and mCherry signals were observed evenly in the cytoplasm and nuclei of the same cells of co‐infiltrated leaves (Figure 3b, upper panel). In addition, expression of the two marker proteins was also monitored in the systemic infected tissues, including the upper leaves, stems and roots at 14 dpi (Figure 3b), whereas fluorescent signals were not observed in plants inoculated with the empty vector (Figure S3). To confirm the accumulation of virus and foreign proteins, we further performed Western blotting assays with specific antibodies to detect the BNYVV‐CP, sGFP and mCherry proteins in systemically infected leaves and roots (Figure 3c). This result obviously demonstrates that BNYVV‐based vectors can mediate co‐expression of two proteins in whole plants. These features provide a possible vector for simultaneous expression of two functional subunits for biological studies in whole plants.

### Simultaneous expression of four recombinant proteins from BNYVV‐based vectors

**Figure 4:**
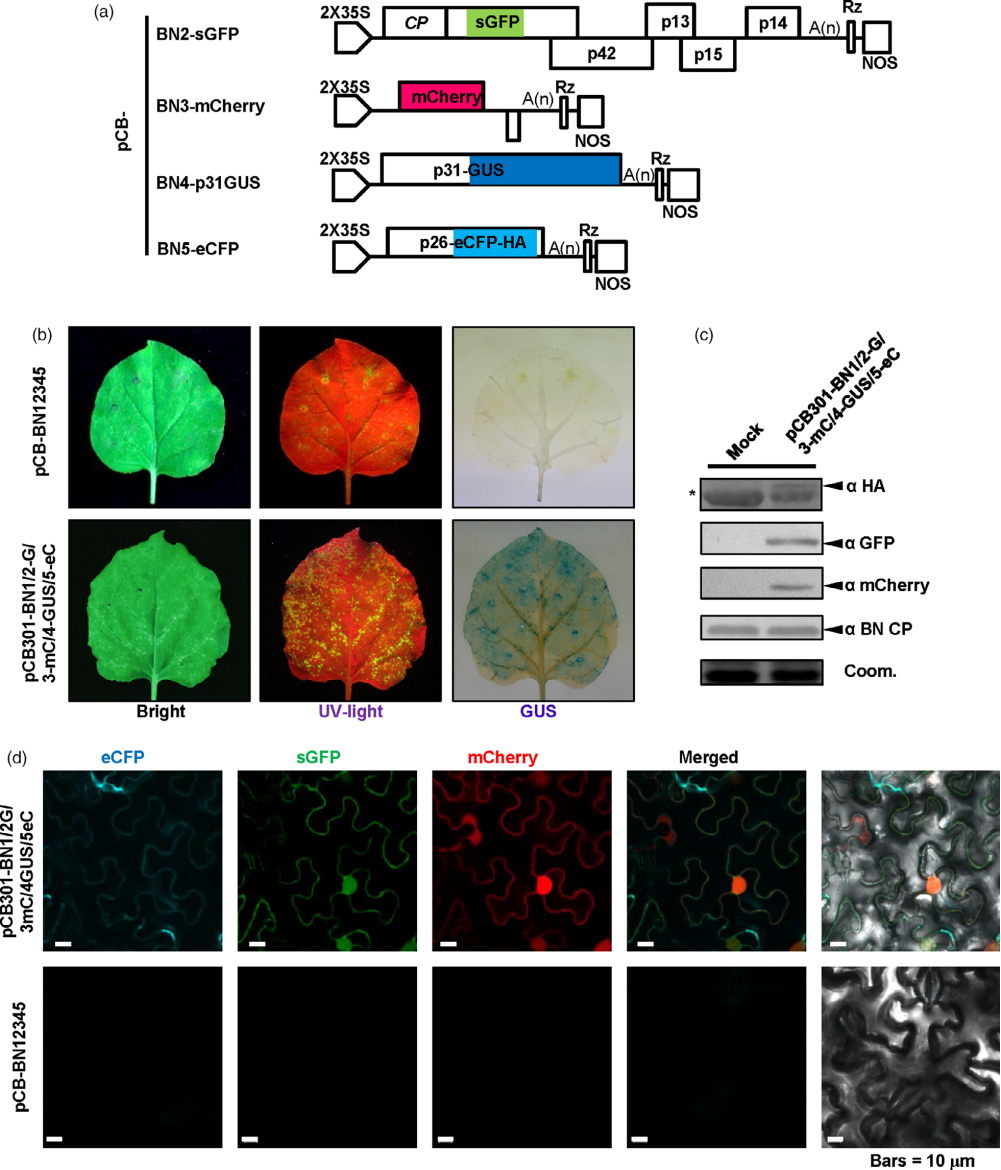
Simultaneous expression of four recombinant proteins from BNYVV‐based vectors in *Nicotiana benthamiana*. (a) Schematic representation of the construction of pCB‐BN2‐sGFP, pCB‐BN3‐mCherry, pCB‐BN4‐p31GUS and pCB‐BN5‐eCFP. The p25 sequence of RNA3 was replaced by mCherry and the eCFP‐HA sequence was fused to the C‐terminus of p26 encoded by RNA5 (b) Symptom expression (Bright), fluorescence observation (UV‐light) and GUS activity (GUS) were detected in *N. benthamiana rdr6*i leaves inoculated with pCB‐BN1/2G/3mC/4GUS/5eC at 5 dpi. The wild type BNYVV infectious clones pCB‐BN12345 was used as a negative control. (c) Immunodetection with corresponding antibodies against BNYVV eCFP‐HA, CP, sGFP and mCherry in infiltrated leaves of *N. benthamiana* infected with pCB‐BN1/2G/3mC/4GUS/5eC. The mock control resulted from pCB‐BN12345 infected plants. The arrow indicates the expected band and the asterisk indicates nonspecific band. Equal loading was verified by staining the gel with Coom. (d) Confocal microscopy of *N. benthamiana* leaves infiltrated with pCB‐BN1/2G/3mC/4GUS/5eC at 5 dpi. Fluorescent signals were not observed in the *N. benthamiana* cells infiltrated with pCB‐BN12345, Bars = 10 μm.

In addition to RNA2 and RNA4, we used Seamless Assembly (Figure S2) to engineer BNYVV RNA 3 and 5 expression vectors. For this purpose, we substituted the mCherry ORF for the p25 ORF of pCB‐BN3 to create pCB‐BN3‐mCherry, and also fused the enhanced cyan fluorescent protein (eCFP) ORF to the C‐terminus of p26 of pCB‐BN5 to generate pCB‐BN5‐eCFP (Figure 4a). Because infections of RNA 3 and RNA 5 are poorly efficient in wild type *N. benthamiana* (Wang *et al*., 2011), we sought to improve BNYVV accumulation by infecting the transgenic line rdr6i, in which the RNA‐dependent RNA polymerase 6 (NbRDR6) antiviral silencing gene is constitutively silenced. Agrobacterium harbouring plasmids pCB‐BN1, pCB‐BN2‐sGFP, pCB‐BN3‐mCherry, pCB‐BN4‐p31GUS and pCB‐BN5‐eCFP (Figure 4a) were mixed and co‐infiltrated into *rdr6*i plants. At 5 dpi, GFP fluorescence expressed from RNA2‐based vector was visualized under long wave UV light in infiltrated leaves (Figure 4b). RNA4‐mediated GUS expression also co‐localized with the sGFP fluorescence elicited by the RNA2‐based vector (Figure 4b, bottom panel).

The leaves were also analysed for eCFP, sGFP and mCherry signal expression at the same time (Figure 4c). Both sGFP and mCherry signals were observed in the cytoplasm and nuclei of the same cells. In contrast, eCFP signals was detected in the cytoplasm but not in the nuclei of these cells, indicating different subcellular localization of the p26eCFP‐HA fusion protein compared with GFP‐p26 of the ‘Pithiviers’ isolate (Link *et al*., 2005). In fact, RNA5 of the Hu isolate did not induce necrotic symptoms like the Pithiviers’ isolate on *C. quinoa*. As a negative control, the plants inoculated with pCB‐BN12345 did not exhibit fluorescence (Figure 4c). The accumulation of virus and foreign fluorescent proteins was further confirmed by Western blotting using BNYVV‐CP, GFP, mCherry and HA specific antibodies (Figure 4d). Collectively, these results demonstrate that the multipartite BNYVV genome provides an attractive system for expression of multiple foreign proteins.

### Application of the BNYVV‐based vector for expression and subcellular co‐localization of multiple‐genes in *Beta vulgaris*

**Figure 5:**
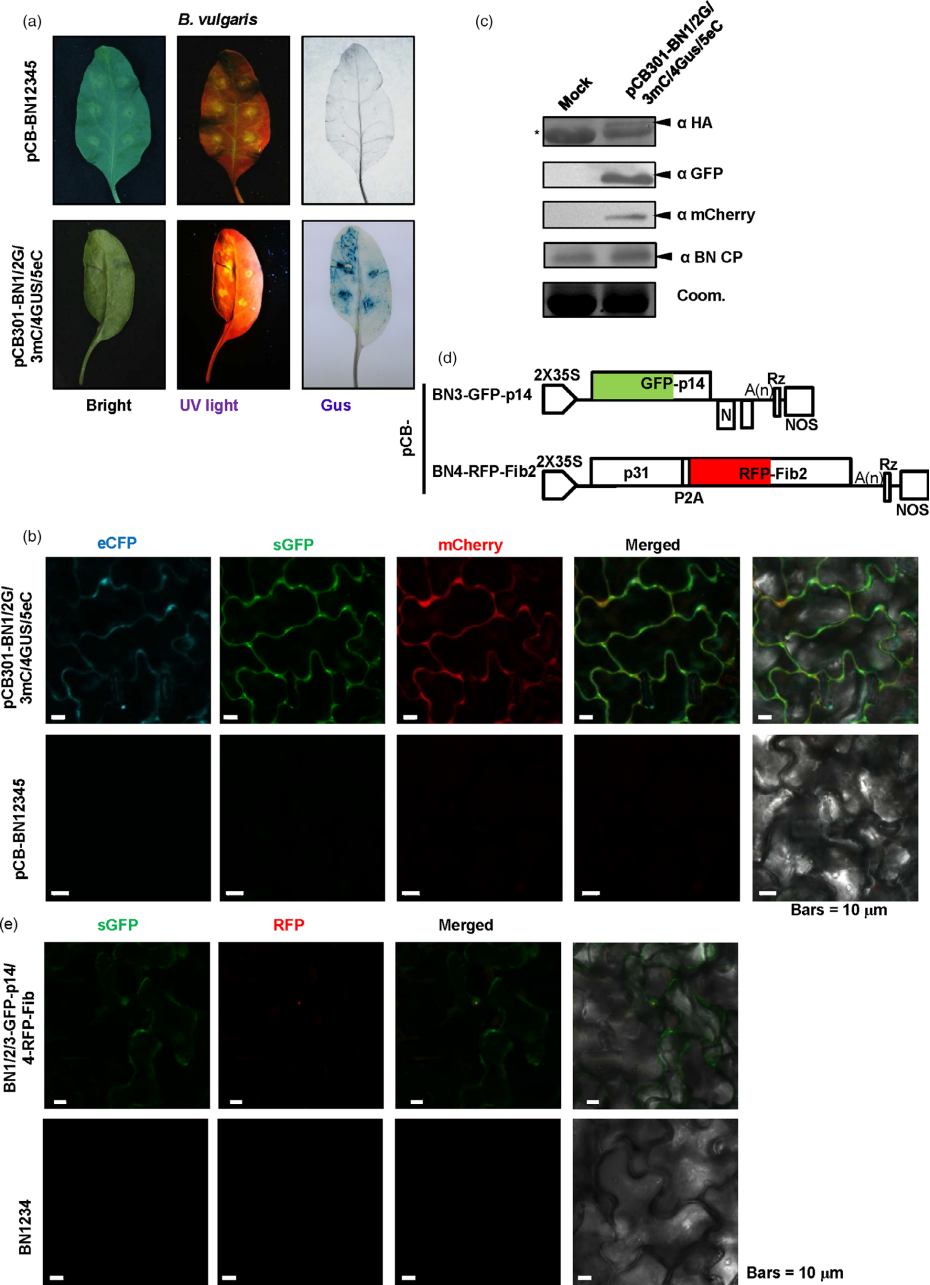
BNYVV‐based vectors for expression and subcellular co‐localization of multiple genes in *Beta vulgaris*. (a) Symptom expression (Bright), fluorescence observation (UV‐light) and GUS activity (GUS) were detected in *B. vulgaris* cv TY‐309 leaves inoculated with pCB‐BN1/2G/3mC/4GUS/5eC at 10 dpi. Wild type BNYVV infectious clones pCB‐BN12345 were used as a negative control. (b) Confocal microscopy of *B. vulgaris* leaves infiltrated with pCB‐BN1/2G/3mC/4GUS/5eC at 8 dpi. Fluorescence was not observed in *B. vulgaris* cells infiltrated with pCB‐BN12345, Bars = 10 μm. (c) Immunodetection using antibodies against BNYVV eCFP‐HA, CP, sGFP and mCherry in infiltrated leaves of *B. vulgaris*. The mock infected plants were agroinfiltrated for expression of the pCB‐BN12345 plasmid. The arrow indicates the expected band and the asterisk indicated a nonspecific band. Equal loading was verified by staining the gel with Coom. (d) Schematic representation of the construction of the pCB‐BN3‐GFP‐p14 and the pCB‐BN4‐RFP‐Fib2 expression vector. The p25 sequence of RNA3 was replaced by the GFP‐p14 sequence. The P2A‐RFP‐NbFib2 sequence was fused to the C‐terminus of RNA4 encoded p31. (e) Confocal images at 10 dpi. showing subcellular distribution of GFP‐p14 and RFP‐Fib2 expressed in *B. vulgaris* cells infiltrated with pCB‐BN1/2/3GFP‐p14/4RFP‐Fib2. Fluorescent signals were not detected in *B. vulgaris* cells infected with the wild type BNYVV pCB‐BN12345 infectious clones. Bars = 10 μm.

Sugar beet (*B. vulgaris*) is an important crop for sugar production, biomass energy and animal feed (Dohm *et al*., 2014; Zakrzewski *et al*., 2017). Thus, it is highly important to carry out genomic studies in sugar beets. However, very few expression vectors have been developed to investigate expression and subcellular co‐localization of proteins in sugar beet cells, so we thus tested the BNYVV‐derived vectors for multiple protein expression and subcellular localization. For this purpose, Agrobacteria strains containing plasmids for pCB‐BN1, pCB‐BN2‐sGFP, pCB‐BN3‐mCherry, pCB‐BN4‐p31GUS and pCB‐BN5‐eCFP (Figure 4a) were mixed and co‐infiltrated into leaves of *B. vulgaris* cv TY‐309. At 7 dpi, the leaves displaying yellowing were analysed for fluorescence signals and GUS expression analysis as described above. Similar to *N. benthamiana*, GFP, mCherry, eCFP signals and GUS expression were detected in the same cells of the infiltrated leaves, but not in the leaves infected by wild type BNYVV (Figure 5a,b). In addition, expression of the coat protein and three fluorescent proteins were all detected by Western blotting using specific antibodies (Figure 5c).

The BNYVV‐based quadripartite vector was also used to investigate subcellular co‐localization in sugar beet cells. In a previous report, the eGFP‐tagged p14 protein accumulated in the nucleoli and the cytoplasm of BY2 cell and *C. quinoa* (Chiba *et al*., 2013), but the subcellular localization of p14 in BNYVV‐infected host sugar beet has not been tested. Hence, we used the RNA3‐based vector to express the GFP‐p14 fusion protein, and the RNA4‐based vector to express RFP‐Fibrillarin (RFP‐Fib2) as a nucleolar marker (Figure 5d). Leaves of TY‐309 were infiltrated with agrobacteria harbouring pCB‐BN1, pCB‐BN2, pCB‐BN3‐GFP‐p14 plus pCB‐BN4‐RFP‐Fib2. At 10 dpi, RFP‐Fib2 signals were localized exclusively in the nucleoli as reported previously (Figure 5e) (Li *et al*., 2018), while GFP‐p14 signals were observed in both the cytoplasm and nucleoli (Figure 5e), suggesting that the cytoplasmic and nucleolar localization of GFP‐p14 on sugar beets is similar to those in BY2 cell and *C. quinoa* (Chiba *et al*., 2013).

These results show that BNYVV‐based vectors are useful for characterization of subcellular co‐localization of multiple‐genes in sugar beet plants.

### BNYVV‐based guide RNA delivery system for CRISPR/Cas9‐mediated plant genome editing

**Figure 6:**
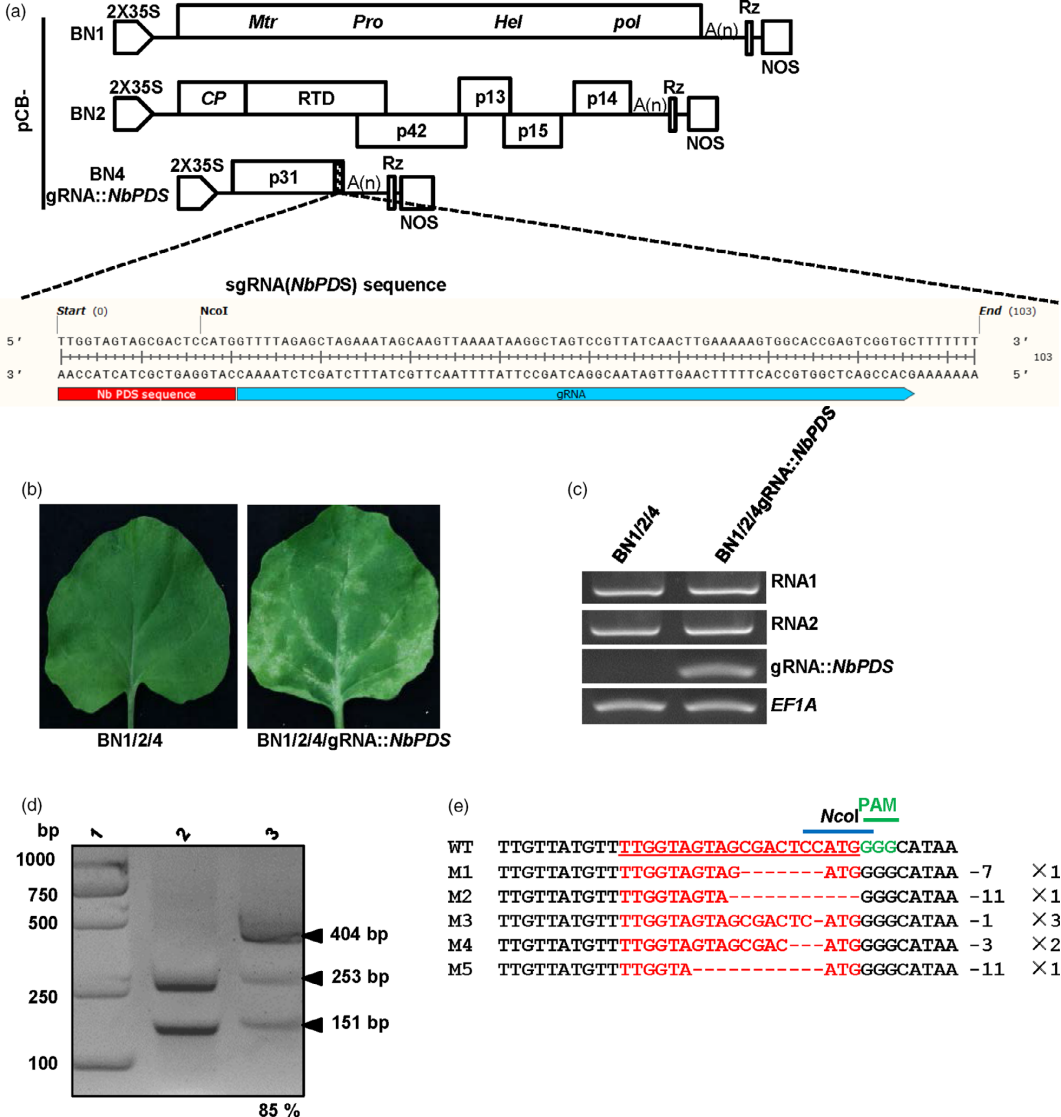
BNYVV‐based genome editing using the CRISPR/Cas9 system in *N. benthamiana*. (a) Schematic representation of the construction of BNYVV‐based genome editing vectors. The *NbPDS3*‐targeting and gRNA scaffold sequences displayed in scale were fused to the C‐terminus of the RNA4 p31 sequence. (b) Photobleached phenotype of transgenic Cas9‐expressing *N. benthamiana* (KQ334) plants inoculated with pCB‐BN1/2/4gRNA::*NbPDS* (right). Lack of photobleaching after agroinfiltration with pCB‐BN1/2/4 (left). Pictures were taken at 5 weeks post‐infiltration (wpi). (c) The infectivity of BNYVV and the expression of gRNA::*NbPDS* were detected in systemic infected leaves of KQ334 plants infiltrated with pCB‐BN1/2/4 or pCB‐BN1/2/4gRNA::*NbPDS* at 5 wpi. *NbEF1A* was used as the control. (d) BNYVV‐based genome editing mutations of *NbPDS* in *N. benthamiana*. The *NbPDS* sequence was amplified by genomic DNA PCR from pCB‐BN1/2/4‐infiltrated control plants or from photobleached leaves of pCB‐BN1/2/4gRNA::*NbPDS*‐infiltrated KQ334 plants. Purified PCR products were digested with *Nco*I and separated on a 2% agarose gel. Lane 2, pCB‐BN1/2/4 infiltrated, line 3, pCB‐BN1/2/4gRNA::*NbPDS* infiltrated. The mutation rate was counted by the software Image J. The arrowheads indicated restriction products. (e): Sanger sequencing of wild type (WT) and mutant versions of *NbPDS* from photobleached leaves area after BNYVV‐based *NbPDS* editing. The target/mutated sequences were shown in red. The protospacer‐associated motif (PAM) is shown in green and the *Nco*I site is shown in blue over the site. Different deletions were indicated by numbers to the right of the sequence (‐ means deletions of n nucleotides).

Although numerous genes have become available in the post‐genomics era, the demand for powerful tools to analyse functions of candidate genes has become an urgent need. The CRISPR/Cas genome editing system has been applied in diverse organisms and has become a valuable technology in plant genetic studies (Lowder *et al*., 2015). To date, efficient delivery of genome editing reagents is necessary for successful genome editing. Plant virus‐based vectors offer attractive systems for high level expression of guide RNAs owning to their efficient replication in host plants (Ali *et al*., 2015a; Yin *et al*., 2015). Here, we further examined whether BNYVV‐based vectors can be used as guide RNA delivery tools in *N. benthamiana* plants. To the end, a gRNA targeting the *N. benthamiana phytoene desaturase* (*NbPDS3*) gene (Figure S4) was cloned and fused to the C‐terminus of the p31 ORF of pCB‐BN4 to generate pCB‐BN4‐gR::*NbPDS* (Figure 6a). Then, Agrobacteria containing pCB‐BN4‐gR::*NbPDS*, or negative control pCB‐BN4, were mixed with Agrobacteria containing pCB‐BN1 and pCB‐BN2 and were infiltrated into the Cas9‐overexpressing *N. benthamiana* plants KQ334 (Yin *et al*., 2015). The systemically infected leaves of *N. benthamiana* plants KQ334 by pCB‐BN4‐gR::*NbPDS* exhibited a photobleaching phenotype at 4 weeks post‐infiltration (wpi), and this phenotype became most visible at 5 wpi (Figure 6b). Three independent experiments were conducted and 78% (26/30) of the pCB‐BN4‐gRNA::*NbPDS*‐infiltrated KQ334 plants developed photobleaching in systemically infected leaves by 5 wpi. In contrast, KQ334 plants infiltrated with pCB‐BN4 did not show the photobleaching phenotype.

To confirm that the photobleaching phenotype was indeed related to genome editing of the *NbPDS3* gene by BNYVV‐based vectors, we further carried out RT‐PCR assays to insure that the *NbPDS*‐targeting gRNAs was expressed in systemic infected leaves by pCB‐BN4‐gR::*NbPDS*, but not by pCB‐BN4 at 5 wpi (Figure 6c). Then, the 404 bp fragment of the *NbPDS3* gene (Ali *et al*., 2015a,2015b) was amplified by genomic DNA PCR from the photobleached areas of leaves from pCB‐BN4‐gR::*NbPDS* or pCB‐BN4‐infiltrated KQ334 plants and was subsequently digested by *Nco*I (Figure 6d). It is striking to note that an *Nco*I site was located within the region of *NbPDS3* targeting by the gRNA sequence (Figure 6a). As expected, the 404 bp fragment of *NbPDS3* gene amplified from the pCB‐BN4‐infected plants was completely digested by *Nco*I into two small DNA bands (Figure 6d, line 2). In contrast, only a small fraction of the *NbPDS3* PCR product from pCB‐BN4gRNA::*NbPDS*‐infected plants could be digested by *Nco*I (Figure 6d, line 3). The band intensity was estimated by ImageJ software, showing that 85% of the *NbPDS3* PCR products from pCB‐BN4‐gR::*NbPDS*‐infected plants were not under digested (Figure 6d, line 3). Then, the PCR products resistant to *Nco*I cleavage were cloned and sequenced. Sequencing alignment showed that five different types of deleted mutations were found in eight clones (Figure 6e), indicating that *NbPDS3* gene editing did occur in KQ334 plants co‐infiltrated with pCB‐BN4‐gR::*NbPDS*. In addition, the different types of sequencing modifications at the target site are shown by sequencing chromatograms, respectively (Figure S5). These results indicate that BNYVV‐based vectors can be used as gRNA delivery tools for efficient genome editing.

## Discussion

Plant virus‐based vectors have been widely used as effective tools for recombinant protein expression and functional genomic studies. However, studies in basic and applied plant biology, such as functional characterization of complexes with heterologous proteins, production of antibodies and pharmaceutical peptides, require simultaneous expression of two or more genes within single cells. Moreover, due to lack of multiple insertion sites, most plant viral vectors with monopartite or bipartite genomes have been unable to express multiple proteins efficiently, although some studies employed multiple copies of the same viral vector backbone to express two proteins simultaneously (Sainsbury *et al*., 2008). Nonetheless, this strategy probably induces superinfection exclusion phenomenon that prevent co‐infections of one viral vector backbone with two different insertions (Giritch *et al*., 2006; Sainsbury *et al*., 2008). Here, we have developed BNYVV isolate Hu (Hu3) infectious cDNA clones and engineered a set of BNYVV‐based gene expression vectors harbouring four insertion sites. Compared with previously BNYVV isolates F13 and Yu2 (Delbianco *et al*., 2013; Laufer *et al*., 2018a), the Hu3 isolate induced much milder symptoms (Figure 2b), which would not affect the phenotypes of inserted foreign proteins. Our current results demonstrate that BNYVV‐based vector can simultaneously express four recombinant proteins in single cells of the infiltration leaves of *N*. *benthamiana* and sugar beet plants (Figures 4 and 5). Additionally, we also observe co‐expression of two reporter proteins in whole *N. benthamiana* plants including the upper leaves, stems and roots (Figure 3).

Another disadvantage of plant virus‐based vectors reported to date is the size constraints and the stability of the recombinant constructs. Although several plant viral vectors can express small foreign genes stably, including as various fluorescent proteins, stably, but expression of larger proteins may be limited because of genome size constraints. However, some studies have shown that *Potexvirus‐*,* Tritimovirus‐*,* Potyvirus‐ and Comovirus*‐derived vectors can express proteins as large as GUS (1.8 kb). However, these vectors usually allow stably expression of only one protein in such situations (Bouton *et al*., 2018; Choi *et al*., 2000; Kelloniemi *et al*., 2008; Zhang *et al*., 2010). BNYVV‐based vector is superior to many existing expression vectors in the aspect of gene‐carrying capacity. Our results demonstrate that BNYVV vectors did not exhibit advantage over other virus expression systems when express single small foreign proteins (Figure S6). However, BNYVV vectors are superior to other vectors in several protein expression aspects and large proteins. In our studies, the set of BNYVV vectors can tolerate insertion sizes from 1104 nt (Figure 5, GFP‐p14) to 2650 nt long (Figure 2, p31GUS). Most strikingly, BNYVV‐based vectors allow simultaneously expression of four proteins in the same cells (Figures 4 and 5), indicating the total insertion size could reach up to 5.8 kb. Therefore, this feature of the BNYVV‐based vectors provides an attractive system for expression of multiple foreign proteins relatively large sizes.

Sugar beet (*B. vulgaris*) is one of the most important crops around the world for sugar production and is also an important plant resource for biomass energy and animal feed (Dohm *et al*., 2014; Zakrzewski *et al*., 2017). Thus, it is highly desirable to identify valuable genes affecting agronomically relevant traits in sugar beet plants. Recently, the sugar beet genome has been sequenced and several transcriptome analyses have identified some important genes related to various biological processes (Dohm *et al*., 2014; Lv *et al*., 2018; Skorupa *et al*., 2016). However, functional characterization of sugar beet genes is still a major challenge due to the difficulty in stable sugar beet transformation. Thus, development of BNYVV‐based vectors will facilitate genomics research in sugar beets and related plants that are susceptible to BNYVV. To date, the reported plant viruses infecting sugar beet plants include BNYVV, BSBMV, *Beet western yellows virus* (Zhou *et al*., 2011), *Beet yellows virus* (Vinogradova *et al*., 2012), *Beet mosaic virus* (Glasa *et al*., 2003) and *Beet curly top virus* (Bach and Jeske, 2014). Nonetheless, previous reports of BNYVV gene expression vectors have only used in *B. macrocarpa*,* C. quinoa* and *N. benthamiana,* but not *B. vulgaris* (Delbianco *et al*., 2013; Schmidlin *et al*., 2005). In this study, we have, for the first time, developed BNYVV‐based vectors that can successfully express four recombinant proteins and have used these vectors for characterization of subcellular co‐localization of multiple‐genes in sugar beet plants (Figure 5). We also demonstrated that BNYVV‐based vectors can be used to deliver gRNA for CRISPR/Cas9 plant genome editing (Figure 6). Compared with currently virus‐based gRNA delivery systems (Ali *et al*., 2015a,2015b, 2018; Yin *et al*., 2015), the efficiency of *NbPDS3* editing by our system is up to 85% (Figure 6d), suggesting BNYVV‐based vectors can be used as efficient gRNA delivery tools for genome editing of sugar beet plants. Thus, the engineering and application of BNYVV‐based expression systems in *Beta* species will provide a valuable platform for insight into functional characterization of sugar beet genes and to promote future sugar beet genome research.

Taken together, our BNYVV‐based vector system combined with other expression strategies could open up new possibilities for co‐expression of a large number of other heterologous proteins of functional significance. In addition, more advanced BNYVV‐based vectors will be developed for functional genomics studies in the future, including virus‐induced gene silencing (VIGS), virus‐based MiRNA expression and virus‐mediated genome editing. In summary, our work provides a convenient and powerful platform for expression of multiple‐genes and for functional characterization of genes in plant molecular biology studies, particularly for an important plant genus that is not easily transformed.

## Experimental procedures

### Plants and virus isolates

*Tetragonia expansa*,* B. vulgaris* susceptible cultivar TY‐309, *B. macrocarpa*, as well as wild type and *rdr6*i *N. benthamiana* were grown for 3–4 weeks at 24 ± 1 °C under a 16 h light and 8 h dark regimen. BNYVV isolates Hu and Hu3 were propagated in *T*. expansa as described previously (Wang *et al*., 2011).

### Construction of full‐length infectious cDNA clones of BNYVV isolate Hu

All the primers used in this work are listed in Table S1.

The full‐length cDNA sequences of BNYVV RNA1 (GenBank: KM434313), RNA2 (GenBank: KM434314) and RNA5 (GenBank: AJ236895.1) were amplified by RT‐PCR using phusion Polymerase (NEB, Beijing, China) from total RNA of Hu isolate of BNYVV (Wang *et al*., 2011). The cDNAs of RNA3 and RNA4 were amplified from the previously T7 driven infectious clones pMDR3 and pUOF1‐6 (Wang *et al*., 2011; Wu *et al*., 2014). The RNA1 and RNA2 cDNA were cloned into the binary vector pCB301‐2x35S‐MCS‐HDV_RZ_‐NOS between the *Stu*I and *Sma*I sites as previously described to generate pCB‐BN1 and pCB‐BN2. In addition, RNAs3, 4 and 5 were individually inserted between the *Stu*I and *Xba*I sites to produce pCB‐BN3, pCB‐BN4 and pCB‐BN5, respectively (Figure 1a). All clones were identified by sequencing and transferred into *A. tumefaciens* strain C58CI.

### Construction of BNYVV‐based vectors

The pCB‐BN2 infectious plasmid was digested with *Kpn*I and *Xba*I at the 1127and 3894 nt sites and the digested vector backbone and the BN2 fragment were purified. Then, the BN2 fragment was inserted into the pUC19‐T vector at the *Kpn*I and *Xba*I sites to produce the pUC‐BN2 intermediate. Inverse‐PCR was performed from pUC‐BN2 using the primer pair BN2‐*Nco*I‐1565F and BN2‐*Xho*I‐1872R resulting in the linearized pCU‐BN2 fragment with *Nco*I *and Xho*I sites replacing the C‐terminal of RTD sequence spanning from nt 1565 to nt 1872. The pUC‐BN2NX construct was obtained by phosphorylating and self‐ligating the linearized pCU‐BN2 fragment. The GFP sequence was amplified with the sGFP‐*Nco*I‐F and sGFP‐*Xho*I‐R primer pair and inserted into the pUC‐BN2NX vector to produce the pUC‐BN2GFP construct. The BN2GFP fragment was recovered by digesting pUC‐BN2GFP with *Kpn*I and *Xba*I, and the released fragment was inserted into the pCB‐BN2GFP backbone produced by *Kpn*I and *Xba*I digestion.

For construction of the RNA3‐derived vector, the entire coding region of p25 was replaced with foreign genes using the easily Seamless Assembly method (Clone Smarter, Houston, USA). To engineer the RNA4‐derived vector, foreign genes were fused to the C‐terminus of p31 with or without the P2A peptide sequence as a linker (Szymczak‐Workman *et al*., 2012), or placed downstream of the first N‐terminal 27 amino acids of p31, which contains the essential *cis‐*acting replication element of RNA4. For construction of the RNA5‐derived vector, foreign genes were fused to the C‐terminus of p26. The details of each construction are described below.

To construct the **pCB‐BN3‐mCherry** clone, the mCherry coding sequence was amplified from pGD‐3G‐mCherry (Sun *et al*., 2018) by PCR using the primer pair R3‐mC‐In‐F and R3‐mC‐In‐R. The purified mCherry fragment was then fused to a linearized vector previously amplified by inverse‐PCR from pCB‐BN3 with the R3Δp25fx‐1 and R3Δp25fx‐2 primers using Seamless Assembly methodology. To construct **pCB‐BN3‐GFP‐P14,** the plasmid pGDG‐p14 was first constructed and the BNYVV p14 fragment was amplified and cloned into the pGDG vector (Goodin *et al*., 2002) to produce pGDG‐p14. The GFP‐p14 fragment was amplified from pGDG‐p14 by PCR using the primer pair G‐P14‐In‐F and G‐P14‐In‐R, and then fused to a linearized vector previously amplified by the inverse‐PCR from pCB‐BN3 with R3Δp25fx‐1 and R3Δp25fx‐2 primers using the Seamless Assembly Kit. To construct **pCB‐BN4P31‐R‐Fib2**, the RFP‐Fibrillin2 fragment was PCR amplified from pGDR‐Fib2 (Li *et al*., 2018) using PCR using the primer pair R‐Fib‐In‐F and R‐Fib‐In‐R, then the purified fragment was fused to a linearized vector previously amplified by the inverse‐PCR from pCB‐BN4 with the P31C‐fx‐1 and P31C‐fx‐2 primers using Seamless Assembly. To construct **pCB‐BN4‐RFP‐Fib2**, P2A was fused between P31 and RFP via inverse‐PCR from pCB‐BN4P31‐R‐Fib2 using the primer pair 31‐2A‐R‐1 and 31‐2A‐R‐2. To construct **pCB‐BN4P31‐P2A‐mCherry,** the mCherry fragment was amplified from pGD‐3G‐mCherry (Sun *et al*., 2018) by PCR using the primer pair 2A‐mC‐In‐F and 2A‐mC‐In‐R and then fused to a linearized vector that had been previously amplified by inverse‐PCR from pCB‐BN4P31‐P2A‐R‐Fib2 with P31‐2A‐fx‐1 and P31C‐2A‐fx‐2 primers by Seamless Assembly. To construct **pCB‐BN4P31‐P2A‐sGFP**, the sGFP fragment was amplified from pGD‐sGFP by PCR using the primer pair 2A‐sG‐In‐F and 2A‐sG‐In‐R and then fused to a linearized vector previously amplified by inverse‐PCR from pCB‐BN4P31‐P2A‐R‐Fib2 with P31‐2A‐fx‐1 and P31‐2A‐fx‐2 primers using Seamless Assembly. To construct **pCB‐BN4P31‐GUS**, the β‐glucuronidase (GUS) ORF was amplified from pUOF6‐1‐GUS (Wu *et al*., 2014) by PCR using the primer pair P31‐GUS‐In‐F and P31‐GUS‐In‐R and then fused to a linearized vector previously amplified by inverse‐PCR from pCB‐BN4 with P31C‐fx‐1 and P31C‐fx‐2 primers using Seamless Assembly. To obtain plasmid **pCB‐BN4P31N27‐GUS**, inverse‐PCR was performed from pCB‐BN4P31‐GUS using the primer pair P31N27‐GUS‐fx‐1 and P31N27‐GUS‐fx‐2. For **pCB‐BN5‐eCFP**, the eCFP‐HA fragment was amplified from pEG102 (Earley *et al*., 2006) by PCR using the primer pair P26‐eC‐HA‐In‐F and P26‐eC‐HA‐In‐R and then fused to a linearized vector previously amplified by inverse‐PCR from pCB‐BN5 with the P26Cfx‐1 and P26Cfx‐2 primers with Seamless Assembly. To construct **pCB‐BN4‐gR::** ***NbPDS***, the gRNA::*NbPDS* sequence was fused to the sequence encoding the C‐terminus of the p31 protein by two rounds of inverse‐PCR. The first inverse‐PCR reaction used pCB‐BN4 and the gRPDS‐31C‐1/2 primer pair. Then, the purified fragment was phosphorylated and self‐ligated to obtain the pCB‐BN4‐gRNbPDS‐1 subclone. The second inverse‐PCR reaction used the same strategy with pCB‐BN4‐gRNbPDS‐1 and the primer pair gRPDS‐31C‐3/4 to generate the final plasmid, **pCB‐BN4‐gR::** ***NbPDS***.

### Plant growth and inoculation

All cDNA clones were transferred into *A. tumefaciens* strain C58CI as described previously (Holsters *et al*., 1978). *Agrobacterium tumefaciens* strains harbouring individual clones were cultured overnight at 28 °C in LB medium containing 25 mg/mL rifampicin and 100 mg/mL kanamycin. Bacterial cells were collected by centrifugation at 4000 ***g*** for 5 min and resuspended in infiltration buffer (0.1 mm acetosyringone, 10 mm MgCl_2_, and 10 mm MES, pH 5.5) to 0.5 OD_600_. Equal amounts of *A. tumefaciens* derivatives were mixed in various combinations and incubated at room temperature for 3–5 h before infiltration. Fully expanded plant leaves were used for agroinfiltration with a needleless syringe.

### Confocal microscopy

Fluorescence signals in viral infected leaves were monitored with a Leica SP8 laser‐scanning microscope (Leica, Wetzlar, Germany). Excitation wavelengths were as follows: eCFP, 430 nm; GFP, 488 nm; RFP, mCherry, 546 nm.

### GUS staining

Infiltrated leaves or systemically infected leaves were harvested for GUS staining as described previously, with minor modifications (Wu *et al*., 2014). Leaves were incubated for 16 h at 37 °C in the darkness within X‐Gluc staining buffer [100 mm sodium phosphate (pH 7.0), 2 mm 5‐bromo‐4‐chloro‐3‐indolyl β‐D‐glucuronic acid, 10 mm EDTA, 0.5 mm potassium ferrocyanide, 0.5 mm potassium ferricyanide and 0.1% Triton X‐100]. Chlorophyll was removed with 70% (v/v) ethanol at room temperature.

### RNA extraction and RT‐PCR detection

Total RNAs of *N. benthamiana* and *B. vulgaris* were extracted from 0.1 g of fresh tissue for RT‐PCR amplification (Wu *et al*., 2014), and cDNAs from 3 μg of total RNA were synthesized in a 30 μL mixture containing M‐MLV Reverse Transcriptase (Promega) and oligo d(T) or BNYVV specific primers as recommended by Promega. Primers used for detection of BNYVV and house‐keeping genes of *N. benthamiana* and *B. vulgaris* are listed in Table S1.

### Protein extraction and Western blotting analysis

Total proteins of *N. benthamiana* or *B. vulgaris* were extracted from 0.1 g of pulverized plant samples in 300 μL of 2 × SDS buffer [100 mm Tris (pH 6.8), 20% glycerol, 10% β‐mercaptoethanol, 4% SDS, and 0.2% bromophenol blue]. Proteins were separated by 12.5% SDS polyacrylamide gel electrophoresis and then transferred to Nitrocellulose Membrane (GE Healthcare, Buckinghamshire, UK). BNYVV CP, p42, and GFP were expressed and purified in *Escherichia coli* and the purified proteins were used to produce polyclonal antibodies in rabbits at the Institute of Genetics and Developmental Biology, Chinese Academy of Sciences. Then, antisera against BNYVV CP (diluted at 1:1000), p42 (diluted at 1:2000), GFP (diluted at 1:2000), mCherry (GenScript, diluted at 1:1000), and HA (Sigma, diluted at 1:1000) were used to detect expression of the relevant proteins with an enhanced chemiluminescence detection kit (GE Healthcare, Buckinghamshire, UK).

### Detection of BNYVV‐based Cas9/gRNA‐mediated mutations in *N. benthamiana* genomic DNA

Genomic DNA was extracted from photobleached leaves of KQ334 *N. benthamiana* plants that had been infiltrated with pCB‐BN1/2/4gRNA::*NbPDS*. Then, 200 ng genomic DNA was used in a PCR reaction containing the NbPDS3‐404 bp‐F and NbPDS3‐404 bp‐R primers and phusion Polymerase (NEB) to amplify the 404 bp NbPDS fragment. The uncut *NbPDS* DNA was purified with a Gel Extraction Kit (Omega, Guangzhou, China), cloned into the pMD19‐T vector (Takara), and DNA sequencing (TsingKe, Beijing) was used to detect mutations.

## Conflicts of interest

The authors declare that there are no conflicts of interest.

## Supporting information


**Figure S1** Local Symptoms in *Nicotiana benthamiana* and *Beta macrocarpa* by agroinfiltration of *Agrobacterium tumefaciens* cells harbouring pCB‐BN1, pCB‐BN2, pCB‐BN3, pCB‐BN4 and pCB‐BN5 (pCB‐BN12345) or the pCB301 empty vector.

**Figure S2** Schematic representation of the construction of BNYVV‐based vectors.

**Figure S3** Confocal images of different *Nicotiana benthamiana* tissues infiltrated with the pCB301 empty vector as a negative control.

**Figure S4** *NbPDS3* target sequence and selected region for genome editing detection.

**Figure S5** Sanger sequencing chromatograms of the indels from photobleached leaf regions.

**Figure S6** Comparison of expression efficiency of foreign protein in different plant viral vectors.

**Table S1** Primers used in this research.
