# Supplementary figures and images for: Development of Beet necrotic yellow vein virus‐based vectors for multiple‐gene expression and guide RNA delivery in plant genome editing
Source: Plant Biotechnol J. 2019 Jan 17;17(7):1302–15. doi: 10.1111/pbi.13055 (PMC6576094; doi:10.1111/pbi.13055)

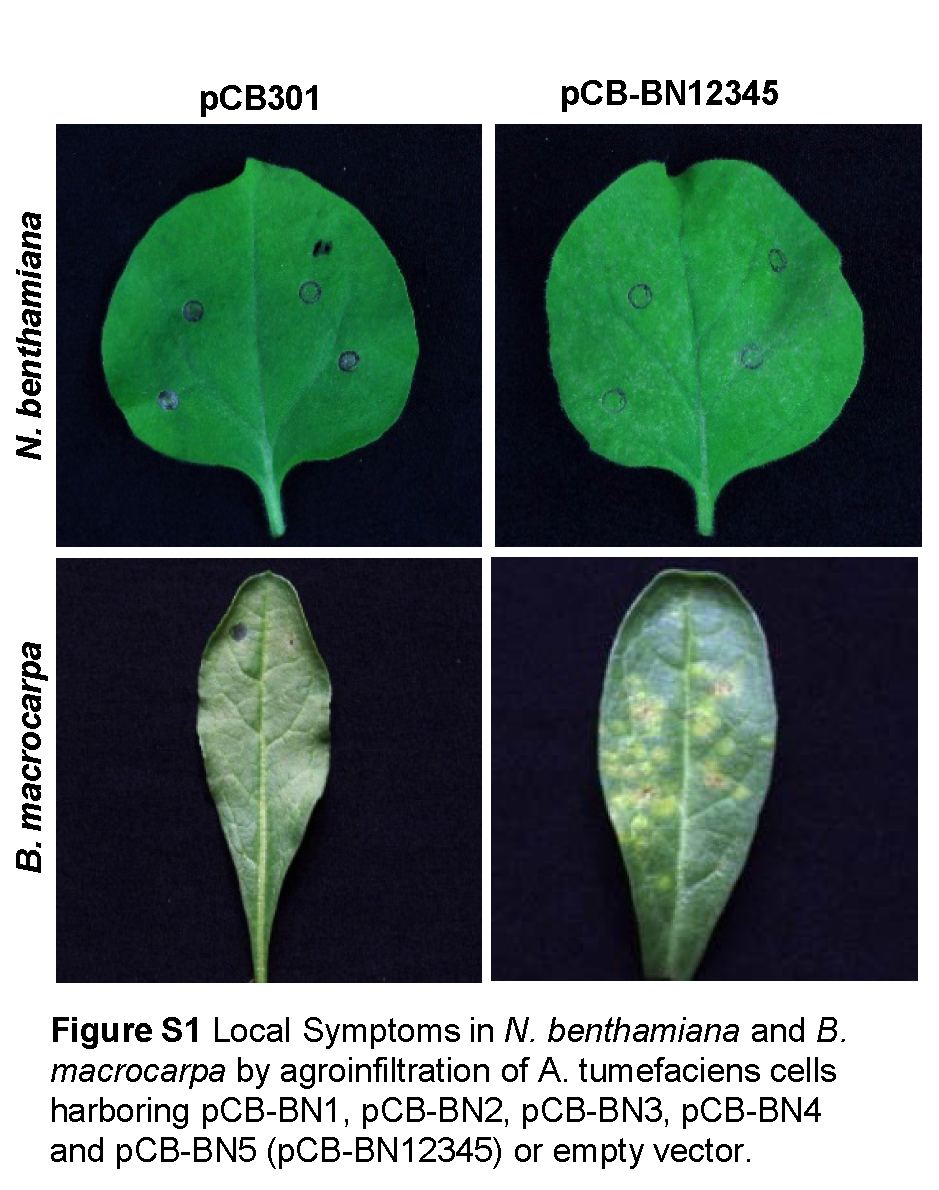

Supplement: Supplementary file 1 — Figure S1 Local Symptoms in Nicotiana benthamiana and Beta macrocarpa by agroinfiltration of Agrobacterium tumefaciens cells harbouring pCB‐BN1, pCB‐BN2, pCB‐BN3, pCB‐BN4 and pCB‐BN5 (pCB‐BN12345) or the pCB301 empty vector. [file PBI-17-1302-s006.tiff]

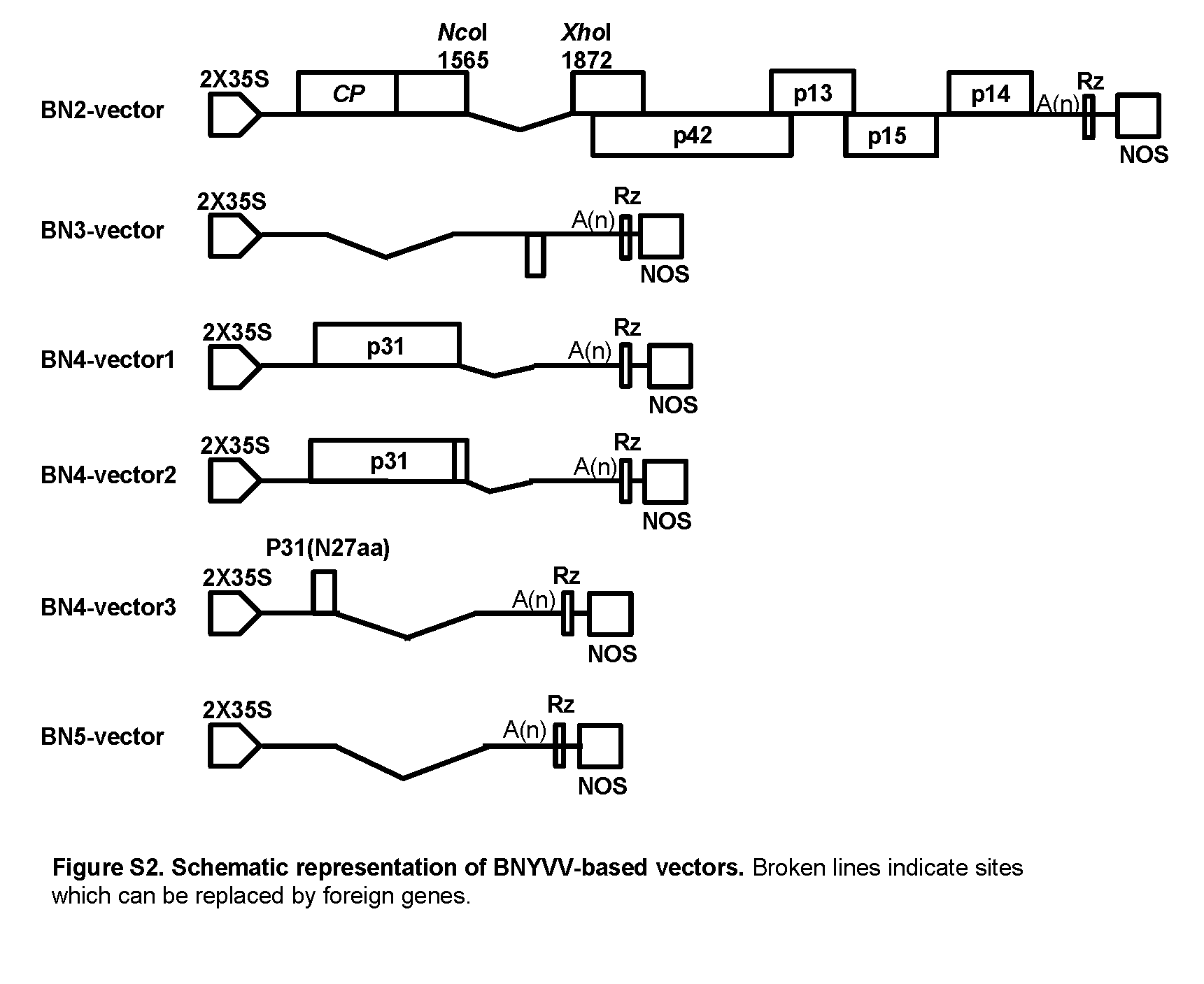

Supplement: Supplementary file 2 — Figure S2 Schematic representation of the construction of BNYVV‐based vectors. [file PBI-17-1302-s005.tiff]

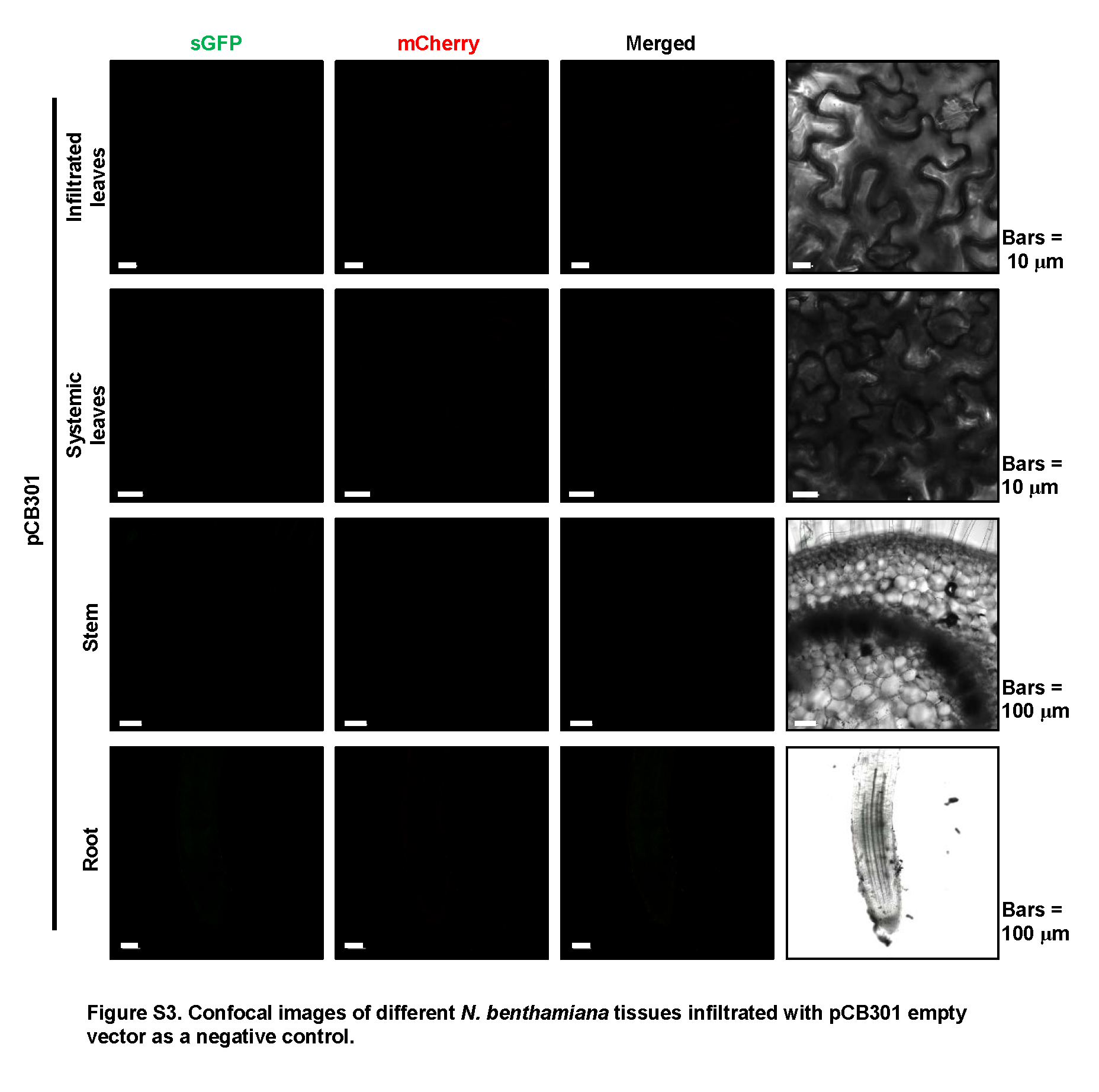

Supplement: Supplementary file 3 — Figure S3 Confocal images of different Nicotiana benthamiana tissues infiltrated with the pCB301 empty vector as a negative control. [file PBI-17-1302-s004.tiff]

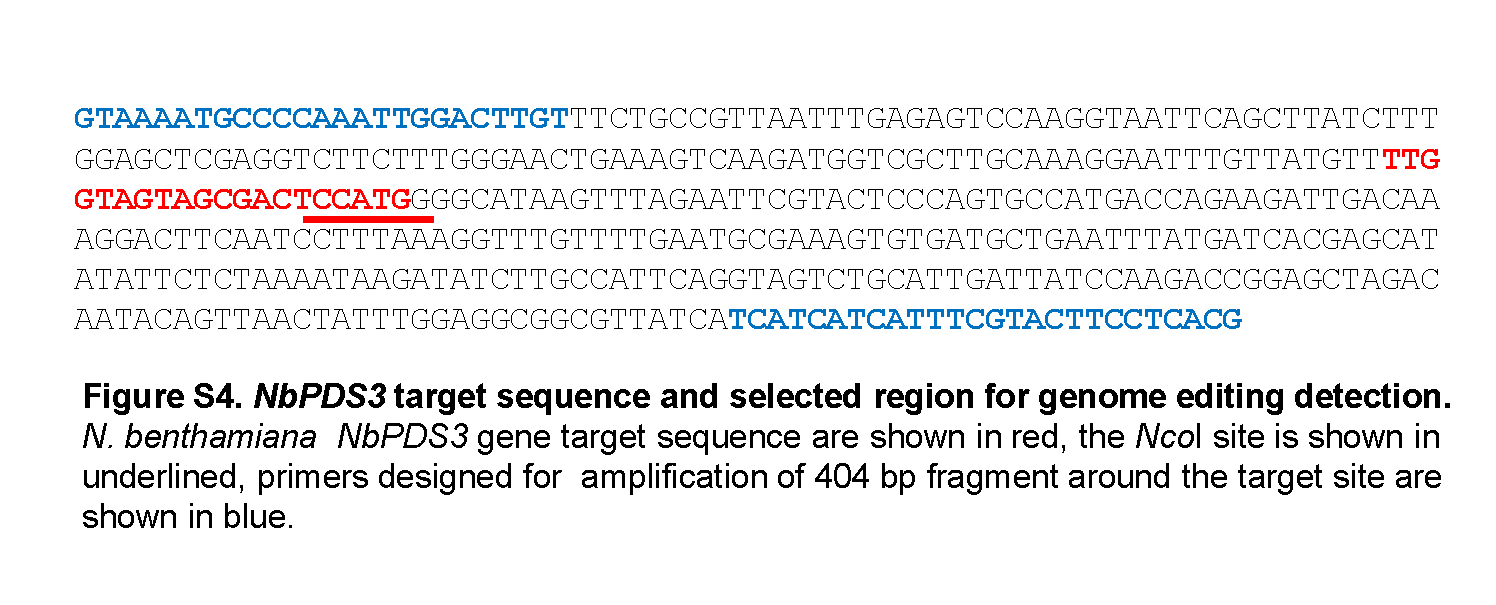

Supplement: Supplementary file 4 — Figure S4 NbPDS3 target sequence and selected region for genome editing detection. [file PBI-17-1302-s007.tiff]

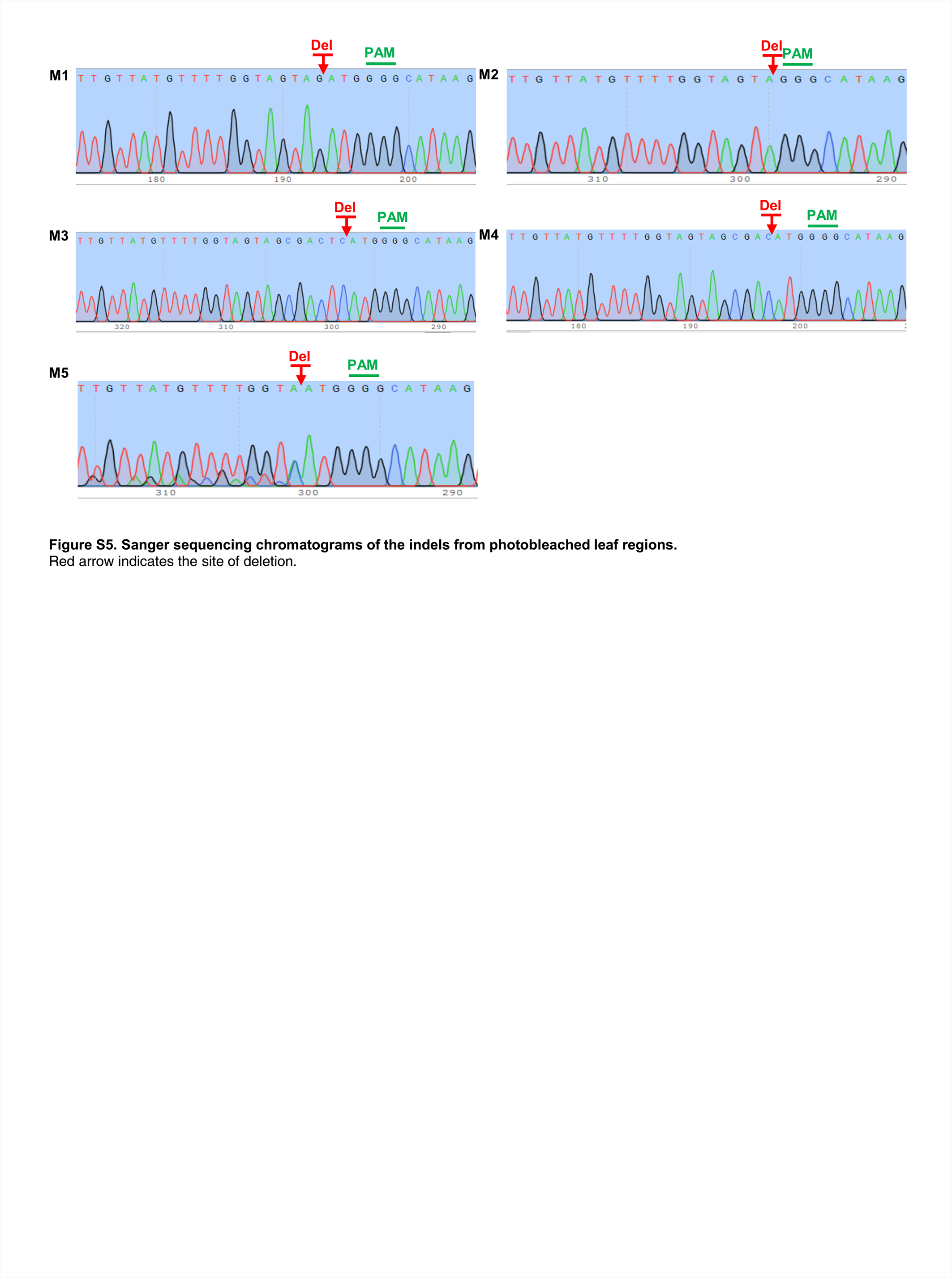

Supplement: Supplementary file 5 — Figure S5 Sanger sequencing chromatograms of the indels from photobleached leaf regions. [file PBI-17-1302-s003.tif]

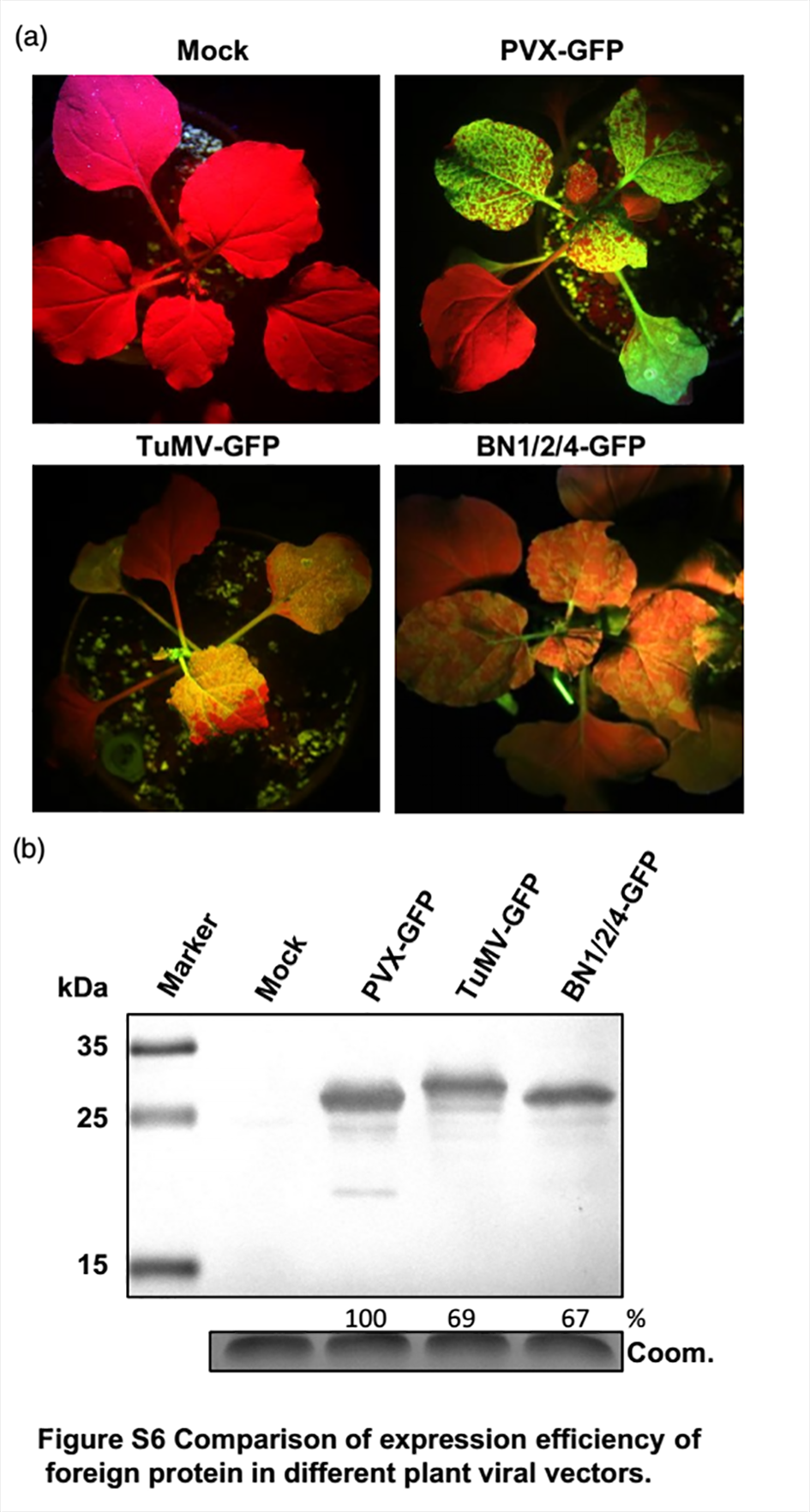

Supplement: Supplementary file 6 — Figure S6 Comparison of expression efficiency of foreign protein in different plant viral vectors. [file PBI-17-1302-s002.tif]
