# Supplementary material for: Development of Beet necrotic yellow vein virus‐based vectors for multiple‐gene expression and guide RNA delivery in plant genome editing
Source: Plant Biotechnol J. 2019 Jan 17;17(7):1302–15. doi: 10.1111/pbi.13055 (PMC6576094; doi:10.1111/pbi.13055)
Supplement: Supplementary file 7 — Table S1 Primers used in this research. [file PBI-17-1302-s001.pdf]

Table. 1 Primers used in this research.

Primers name	Sequence(5’-3’)	Descriptions
BN2-*Nco* I-1565F	CATGCCATGGCATCTCGCTCTCGTCGTTC	To construct pCB-BN2GFP
BN2-*Xho* I-1872R	CCGCTCGAGATGCTGCGACGTGGG	To construct pCB-BN2GFP
sGFP-*Nco* I-F	CATGCCATGGCAAGTAAAGGAGAAGAACT	To construct pCB-BN2GFP
sGFP-*Xho* I-R	CCGCTCGAGTTTGTATAGTTCATCCATGC	To construct pCB-BN2GFP
R3-mC-In-F	TGATCATCATTAAGTGACCGTCATGGTGAGCAAGGGCGAGGAGGATAACATGGCCATC	To construct pCB-BN3△P25-mCherry
R3-mC-In-R	TTGTTGAAATTGTGATAACTCTATCCTCCGCCCTTGTACAGCTCGTCCATGCCGCCGGT	To construct pCB-BN3△P25-mCherry
R3△p25fx-1	TAGAGTTATCACAATTTCAACAACAC	To construct pCB-BN3△P25-mCherry and pCB-BN3△P25-G-P14
R3△p25fx-2	GACGGTCACTTAATGATGATCAGAA	To construct pCB-BN3△P25-mCherry and pCB-BN3△P25-G-P14
G-P14-In-F	TGATCATCATTAAGTGACCGTCATGGGTAAAGGAGAAGAACTTTTC	To construct pCB-BN3△P25-G-P14
G-P14-In-R	TGTTGTTGAAATTGTGATAACTCTACACCTCAGGATCGACAATAACA	To construct pCB-BN3△P25-G-P14
R-Fib-In-F	GTGGTTTGTC TTTTATCACGATATGGCCTCCTCCGAGAACGTCATC	To construct pCB-BN4P31-P2A-R-Fib2
R-Fib-In-R	AAACACATCATAAATACCACTAGGCAGCAGCCTTTTGCTTCTTCGG	To construct pCB-BN4P31-P2A-R-Fib2
P31C-fx-1	TGGTATTTATGATGTGTTTAGTTATTCG	To construct pCB-BN4P31-P2A-R-Fib2 and pCB-BN4P31-Gus
P31C-fx-2	ATCGTGATAAAAGACAAACCACAGAAAG	To construct pCB-BN4P31-P2A-R-Fib2 and pCB-BN4P31-Gus
31-2A-R-1	GCAAGCAGGAGACGTGGAAGAAAACCCCGGTCCTATGGCCTCCTCCGAGAACGTCATCA	To construct pCB-BN4P31-P2A-R-Fib2
31-2A-R-2	TTTAACAGAGAGAAGTTCGT GGCTCCGGATCCCATATCGTGATAAAAGACAAACCACAG	To construct pCB-BN4P31-P2A-R-Fib2
2A-mC-In-F	AGACGTGGAAGAAAACCCCGGTCCTATGGTGAGCAAGGGCGAGGAGGATAACATG	To construct pCB-BN4P31-P2A-mCherry
2A-mC-In-R	ATAACTAAACACATCATAAATACCATTACTTGTACAGCTCGTCCATGCCGCCGGT	To construct pCB-BN4P31-P2A-mCherry
P31-2A-fx-1	TGGTATTTATGATGTGTTTAGTTATTCGAG	To construct pCB-BN4P31-P2A-mCherry
P31-2A-fx-2	AGGACCGGGGTTTTCTTCCACGTCTCCTGC	To construct pCB-BN4P31-P2A-mCherry
P31-Gus-In-F	TCTGTGGTTTGTCTTTTATCACGATATGTTACGTCCTGTAGAAACCCCAA	To construct pCB-BN4P31-Gus
P31-Gus-In-R	ATAACTAAACACATCATAAATACCACTATTGTTTGCCTCCCTGCTGCGGT	To construct pCB-BN4P31-Gus
P31N27-Gus-fx-1	ATGTTACGTCCTGTAGAAACCCCAACCCGT	To construct pCB-BN4P31$_{N27}$-Gus
P31N27-Gus-fx-2	CCTAGCAGTACAATCATAGTCTTCATGACG	To construct pCB-BN4P31$_{N27}$-Gus
P26-eC-HA-In-F	CATGATAATGATTGTGGAGGCGGAGGAATGGTGAGCAAGGGCGAGGAGCTGTTC	To construct pCB-BN5P26-eCFP-HA
P26-eC-HA-In-R	TAACGTCTTCGGACGTCACAAACGATTAAGCGTAATCTGGAACATCGTATGGGTA	To construct pCB-BN5P26-eCFP-HA
P26Cfx-1	TCGTTTGTGACGTCCGAAGACG	To construct pCB-BN5P26-eCFP-HA
P26Cfx-2	TCCACAATCATTATCATGATCT	To construct pCB-BN5P26-eCFP-HA

Primers name	Sequence(5’-3’)	Descriptions
gRPDS-31C-1	AAAGTGGCACCGAGTCGGTGCTTTTTTTTGGTATTTATGATGTGTTTAGTTATTCGAG	To construct pCB-BN4-gR:PDS
gRPDS-31C-2	TCTAAAACCATGGAGTCGCTACTACCAACTAATCGTGATAAAAGACAAACCACAGAAAG	To construct pCB-BN4-gR:PDS
gRPDS-31C-3	AGGCTAGTCCGTTATCAACTTGAAAAAGTGGCACCGAGTCGGTGCTTTTTTT	To construct pCB-BN4-gR:PDS
gRPDS-31C-4	TATTTTAACTTGCTATTTCTAGCTCTAAAACCATGGAGTCGCTACTACCA A	To construct pCB-BN4-gR:PDS
BN1-2247F	GGCAGATAGGTTGAAGGCTAATG	For RT-PCR detection of BNYVV RNA1
BN1-2802R	AAATGTATACATAGACTCAG	For RT-PCR detection of BNYVV RNA1
BNCPF	CGGGGTACCCGATGTCGAGTGAAGGTAGATA	For RT-PCR detection of BNYVV RNA2
BNCPR	GCTCTAGACTATTGTCCGGGTGGACTGG	For RT-PCR detection of BNYVV RNA2
P25F	TATCAAGTTGTTGTGTTTTCTG	For RT-PCR detection of BNYVV RNA3
P25R	TATGGGAACATTATGAACACG	For RT-PCR detection of BNYVV RNA3
P31F	ATGGCTGATGGAGAGATATG	For RT-PCR detection of BNYVV RNA4
P31R	CTAATCGTGATAAAAGACAA	For RT-PCR detection of BNYVV RNA4
P26F	ATGGATATTGATCATTGTAT	For RT-PCR detection of BNYVV RNA5
P26R	TCATCCACAATCATTATCAT	For RT-PCR detection of BNYVV RNA5
BvActin-F	GGCAAACAGGGAAAAGATGA	For RT-PCR detection
BvActin-R	ACGACCAGCAAGATCCAAAC	For RT-PCR detection
NbEF1A-F	AGCTTTACCTCCCAAGTCATC	For RT-PCR detection
NbEF1A-R	AGAACGCCTGTCAATCTTGG	For RT-PCR detection
PDSgR-F	TTGGTAGTAGCGACTCCATG	For RT-PCR detection of gRNA：PDS
PDSgR-R	GCACCGACTC GGTGCCACTTT	For RT-PCR detection of gRNA：PDS
NbPDS3-404bp-F	GTAAAATGCCCCAAATTGGACTTGT	For amplifying 404 bp fragment around NbPDS target site
NbPDS3-404bp-R	CGTGAGGAAGTACGAAATGATGATGA	For amplifying 404 bp fragment around NbPDS target site
2A-sG-In-F	TGGAAGAAAACCCCGGTCCTATGGCAAGTA AAGGAGAAGA ACTTTTCACT	To construct pCB-BN4P31-P2A-sGFP
2A-sG-In-R	TAAACACATCATAAATACCATTATTTGTAT AGTTCATCCA TGCCATGTGT	To construct pCB-BN4P31-P2A-sGFP
